# MCTR1 enhances the resolution of lipopolysaccharide‐induced lung injury through STAT6‐mediated resident M2 alveolar macrophage polarization in mice

**DOI:** 10.1111/jcmm.15481

**Published:** 2020-08-05

**Authors:** Qian Wang, Hua‐Wei Zhang, Hong‐Xia Mei, Yang Ye, Hao‐Ran Xu, Shu‐Yang Xiang, Qian Yang, Sheng‐Xing Zheng, Fang‐Gao Smith, Sheng‐Wei Jin

**Affiliations:** ^1^ Department of Anesthesia and Critical Care The Second Affiliated Hospital and Yuying Children’s Hospital of Wenzhou Medical University Zhejiang China; ^2^ Institute of Inflammation and Aging College of Medical and Dental Sciences University of Birmingham Birmingham UK

**Keywords:** acute respiratory distress syndrome, alveolar macrophage, lipopolysaccharide, MCTR1, resolution phase of inflammation

## Abstract

Acute respiratory distress syndrome (ARDS) is a fatal disease characterized by excessive infiltration of inflammatory cells. MCTR1 is an endogenously pro‐resolution lipid mediator. We tested the hypothesis that MCTR1 accelerates inflammation resolution through resident M2 alveolar macrophage polarization. The mice received MCTR1 via intraperitoneal administration 3 days after LPS stimulation, and then, the bronchoalveolar lavage (BAL) fluid was collected 24 hours later to measure the neutrophil numbers. Flow cytometry was used to sort the resident and recruited macrophages. Post‐treatment with MCTR1 offered dramatic benefits in the resolution phase of LPS‐induced lung injury, including decreased neutrophil numbers, reduced BAL fluid protein and albumin concentrations and reduced histological injury. In addition, the expression of the M2 markers Arg1, FIZZ1, Remlα, CD206 and Dectin‐1 was increased on resident macrophages in the LPS + MCTR1 group. Resident macrophage depletion abrogated the therapeutic effects of MCTR1, and reinjection of the sorted resident macrophages into the lung decreased neutrophil numbers. Finally, treatment with MCTR1 increased STAT6 phosphorylation. The STAT6 inhibitor AS1517499 abolished the beneficial effects of MCTR1. In conclusion, MCTR1 promotes resident M2 alveolar macrophage polarization via the STAT6 pathway to accelerate resolution of LPS‐induced lung injury.

## 1 INTRODUCTION

Acute respiratory distress syndrome (ARDS) is a devastating clinical syndrome which characterized by an uncontrolled pulmonary inflammation and inflammatory cell accumulation.^1^, ^2^ Investigation of experimental ARDS models has mainly focused on the early inflammation phase,^3^ but this strategy has limitations in clinical applications. In contrast, a focus on regulating the resolution phase may provide a new strategy for treatment of ARDS.^3^ A previous study indicated that macrophages were necessary to address pulmonary inflammation.^4^ Pulmonary inflammation is closely related to two phenotypically distinct cell populations‐resident and recruited macrophages.^5^, ^6^, ^7^ During embryogenesis and self‐renew throughout life, resident macrophages accumulate in the lungs^8^, ^9^; recruited macrophages are derive from circulating monocytes, which are transported to the site of inflammation and then mature into macrophages. A recent study showed that resident macrophages suppressed, whereas recruited macrophages promoted, allergic pulmonary inflammation.^10^ Another study demonstrated that resident macrophages were not involved in cytokine production, but recruited macrophages seem to be preparative for inflammatory signals with arginine metabolism and cytokine.^11^ Therefore, the two populations of macrophages exhibit different capabilities in inflammatory lung disease.

Macrophages can be subclassified into M1 (classically activated or pro‐inflammatory) and M2 (alternatively activated or anti‐inflammatory) phenotypes.^6^, ^7^, ^12^ M1 macrophages secrete pro‐inflammatory cytokines, which are detrimental to wound healing.^13^ M2 macrophages are involved in tissue regeneration, growth, angiogenesis and matrix wound healing, thereby supporting tissue remodelling.^14^, ^15^, ^16^ Previous studies demonstrated that macrophages transform from a pro‐inflammatory M1 phenotype to a pro‐resolution M2 phenotype during acute inflammation, which initiates lung repair and restores tissue homoeostasis.^17^, ^18^ Thus, macrophage polarization is important in accelerating the resolution of inflammation and maintaining tissue homoeostasis.

Maresin conjugates in tissue regeneration (MCTR) are a new series of lipid mediators.^19^, ^20^ MCTR1 (13‐glutathionyl, 14‐hydroxydocosahexaenoic acid) is obtained from human macrophages and sepsis patients^21^ and regulates processes associated with the inflammation‐resolution phase, tissue repair and regeneration.^22^, ^23^ MCTR1 not only significantly reduced neutrophil accumulation, but also obviously stimulated macrophage efferocytosis, a key pro‐resolution action.^24^, ^25^

We hypothesis that treatment with MCTR1 at the peak of inflammation promotes the resolution of inflammation in LPS‐induced lung injury. Our secondary hypothesis was that accelerated inflammation resolution is associated with resident M2 macrophage polarization. Finally, by investigating the downstream signalling pathways of MCTR1, we determined the effect of STAT6 on resident M2 alveolar macrophage polarization to gain a better understanding of the mechanisms.

## 2 MATERIALS AND METHODS

### 2.1 Materials

MCTR1 was from Cayman Chemical Company. AS1517499 was purchased from MedChemExpress. LPS (*Escherichia coli* serotype 055:B5) was from Sigma. F4/80‐PE‐Cyanine7, CD11c‐PerCP‐Cyanine5.5, CD11b‐APC, CD206‐PE, Ly‐6G‐FITC, CD86‐PE, Dectin‐1‐PE, anti‐P‐STAT6 and total STAT6 (T‐STAT6) antibodies were from Invitrogen. Anti‐Relmα, Arg‐1 and Ym‐1 antibodies were from Abcam.

### 2.2 Animal preparation

C57BL/6 mice (20‐25 g) were from Slac Laboratory Animal. The study was approved by the Animal Studies Ethics Committee of Wenzhou Medical University.

Atomization inhalation of LPS (1 mg/kg) is to establish lung injury model. In MCTR1 group, mice received MCTR1 (0.1 µg/mouse) via intraperitoneal administration. In LPS + MCTR1 group, the mice received MCTR1 via intraperitoneal injection 3 days after LPS stimulation. For the LPS + MCTR1+AS1517499 group, the mice received AS1517499 (10 mg/kg) via intraperitoneal administration 2 hours before MCTR1 intervention. After anesthetization with sodium pentobarbital (60 mg/kg, i.p.), BAL fluid was collected on Day 4.

### 2.3 Pathological studies

For pathological studies, the trachea was cannulated with a tube, and the lungs were infused with 4% paraformaldehyde at 25 cm H2O (2.4 kPa) for 1 hour. The lungs were removed with the trachea securely tied with surgical sutures under a pressure of 25 cm H2O, fixed in 4% paraformaldehyde for an additional 24 hours, embedded in paraffin and stained with haematoxylin and eosin for light microscopy analysis. Acute lung injury score was based on alveolar congestion, alveolar haemorrhage, alveolar wall/hyaline membrane thickness and inflammatory cell accumulation. 0 = no injury; 1 = slight injury (25%); 2 = moderate injury (50%); 3 = severe injury (75%); and 4 = very severe injury (almost 100%).

### 2.4 Flow cytometry

BAL cells were cultured with variety antibodies for 20 minutes and FACS lysing solution for 10 minutes, then centrifuged at 400 *g* for 5 minutes and resuspended in PBS to analyse using FACSCalibur (Beckman Coulter). Resident macrophages were defined as F4/80^+^CD11b^‐^CD11c^+^, and recruited macrophages were labelled as F4/80^+^CD11b^+^CD11c^‐^ as previously described.^26^

### 2.5 Resident macrophages depletion

Clodronate liposomes (5 mg/mL, 50 μL) were intratracheal instillation to deplete the resident macrophage. PBS liposomes were used as control. Three days later, the mice underwent aerosol inhalation with LPS (1 mg/kg). After another 3 days, the mice received MCTR1 via intraperitoneal administration. Then, BAL fluid was collected.

### 2.6 Depletion of recruited macrophages

Recruited macrophages were depleted by a single caudal vein injection of clodronate liposomes (200 μL) 8 hours before the administration of LPS. Three days after 1 mg/kg LPS administration, the mice received MCTR1 via intraperitoneal injection. Then, BAL fluid was harvested.

### 2.7 Cell sorting and reinjection

Cells were sorted using a 100 μm nozzle size and then directly into tubes containing RPMI by a CytoFLEX flow cytometer (Beckman Coulter). The sorted resident or recruited macrophages (1 × 10^6^) were reinjected intratracheally into the lung. The mice were randomized into five groups (n = 6‐8): LPS group, recruited Mø group, resident Mø group, MCTR1 recruited Mø group and MCTR1 resident Mø group. The resident and recruited macrophages were sorted after LPS stimulation for 3 days. For the recruited, resident Mø and LPS groups, the mice received sorted resident macrophages, recruited macrophages or an equivalent volume of PBS via intratracheal injection. Next, MCTR1 was administered on Day 3 after LPS administration, and resident and recruited macrophages were sorted on Day 4. Then, the cells were reinjected into the lung to establish the MCTR1 resident Mø and resident Mø groups. Twenty‐four hours later, BAL fluid was collected for neutrophil count analysis.

### 2.8 Western blotting

Proteins were extracted using RIPA lysis buffer, and protein concentration was measured by a protein assay kit. 10% sodium‐dodecyl sulphonate polyacrylamide gel was used to separate the protein. After primary and secondary antibodies cultured, the protein bands were measured by using a UVP gel imaging system.

### 2.9 ELISA

The BAL fluid from individual mice was centrifuged, and the supernatant was collected. The protein, albumin, IL‐4, IL‐13 and IL‐1β levels in the BAL fluid were determined. Furthermore, IL‐4, IL‐13 and IL‐1β concentrations in the lung homogenate were also detected by ELISA (R&D Systems).

### 2.10 Statistical analysis

The data were expressed as the mean ± SD. All data were analysed by one‐way ANOVA followed by Tukey's post hoc test for multiple comparisons. Significance was determined at *P* < .05. Statistical analyses were performed using Prism 6.0 software.

## 3 RESULTS

### 3.1 MCTR1 accelerated the resolution of inflammation in LPS‐induced lung injury

**FIGURE 1:**
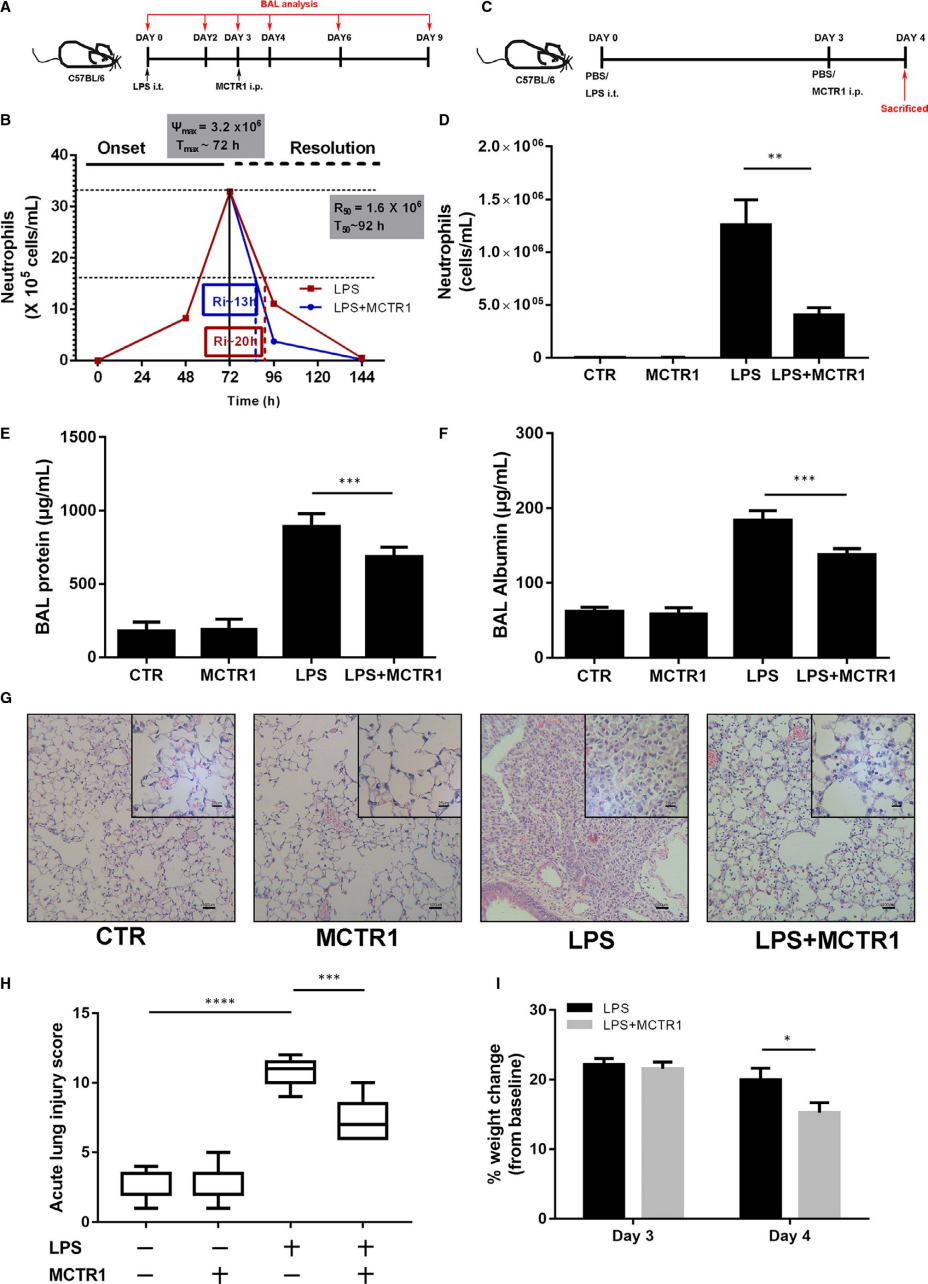
MCTR1 accelerated the resolution of inflammation in LPS‐induced acute lung injury. Mice received an intratracheal atomization of 1 mg/kg LPS and then were injected i.p. with MCTR1 (0.1 µg/mouse) 3 d later. The BAL fluid was collected on Day 4, Day 6 and Day 9 (A). The resolution of inflammation was defined in operative and quantitative terms by the following resolution indices: 1) magnitude (Ψ_max_, *T*_max_), the time‐point (*T*_max_) at which neutrophil numbers reached a maximum (Ψ_max_); 2) duration (*R*_50_, *T*_50_), the time‐point (*T*_50_) at which the neutrophil numbers were reduced to 50% of Ψmax (*R*_50_); 3) Ri, the time interval from the maximum neutrophil point (Ψ_max_) to the 50% reduction point (*R*_50_) (ie *T*_50_‐*T*_max_) (B). The effects of MCTR1 were assessed on Day 4 by neutrophil count via flow cytometry (C, D), BAL protein (E) and BAL albumin (F) expression levels by ELISA, haematoxylin and eosin staining (original magnification: ×100, ×400) (G), acute lung injury score (H) and weight recovery (%weight change = (baseline‐present)/baseline) (I). The data are presented as the mean ± SEM. n = 6‐8. *P < .05, **P < .01, ***P < .001, ****P < .000

The experiment was designed as indicated in Figure 1A. We first determined neutrophil changes by using an LPS‐induced lung injury model. Neutrophil numbers peaked at 72 hours and then gradually decreased until 144 hours (Day 6). Between 72 hours (*T*_max_) and 92 hours (*T*_50_), total neutrophil numbers reduced from 3.2 × 10^6^ (Ψ_max_; maximal PMN number) to 1.6 × 10^6^ (*R*_50_; 50% reduction in neutrophils). For a direct comparison, this period of neutrophilic loss from the exudates (ie 13‐20 hours) is termed the resolution interval (Ri) (Figure 1B). MCTR1 reduced the *T*_50_ from 92 to 85 hours, thus shortening the Ri from 20 to 13 hours compared with that of the LPS group (Figure 1B).

The number of neutrophils decreased most quickly on Day 4 after administration of MCTR1 compared with that of the LPS group (Figure 1C,D). In addition, both the protein and albumin concentrations in the BAL fluid were reduced in the LPS + MCTR1 group compared with those of the LPS group (*P* < .05; Figure 1E,F). However, there was no significant difference between the CTR and MCTR1 groups (Figure 1D‐F; *P* > .05).

Furthermore, the lung architecture in the LPS group was obviously damaged, with inflammatory cell infiltration, interstitial oedema, damaged pulmonary alveoli structures and haemorrhaging. All morphological changes were less pronounced in the LPS + MCTR1 group (Figure 1G). However, there was no significant difference between the CTR and MCTR1 groups (*P* > .05) (Figure 1G). Acute lung injury scores were quantified in parallel with the pathophysiological changes (Figure 1H). The bodyweight recovered faster in the LPS + MCTR1 group than in the LPS group on Day 4 (*P* < .05) but not on Day 3 (*P* > .05; Figure 1I). (%weight change = (baseline‐present)/baseline).

### 3.2 MCTR1 enhanced M2 macrophage polarization

**FIGURE 2:**
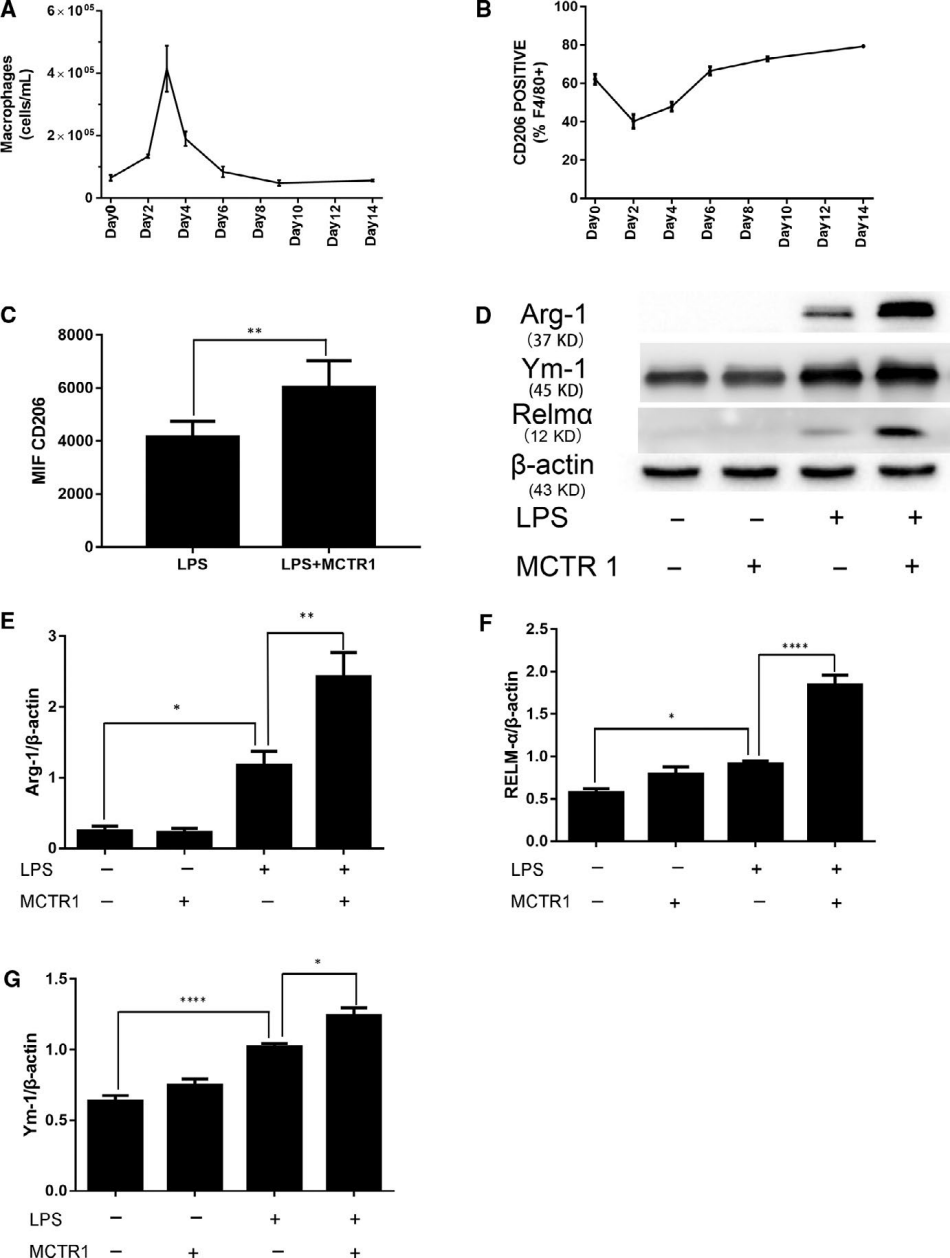
MCTR1 promoted M2 macrophage polarization in LPS‐induced acute lung injury. Macrophages in the BAL fluid were counted by flow cytometry after 1 mg/kg LPS was administered by inhalation (A). Next, CD206‐positive cells (M2 macrophages) were measured (B). Then, MCTR1 (0.1 µg/mouse) was administered 3 d after LPS (1 mg/kg) stimulation, and the MIF of CD206 (C), Arg‐1, Ym‐1 and Relma (M2 marker) expression in the lung tissue homogenate on Day 4 was measured (D‐G). The data are presented as the mean ± SEM. n = 6‐8. *P < .05, **P < .01, ****P < .000

The number of macrophages in BAL fluid increased and peaked on Day 3 in LPS‐induced lung injury (Figure 2A). CD206 expression in BAL macrophages was decreased on Day 2 and then gradually increased until Day 14 after LPS administration (Figure 2B). The macrophages were most prominently decreased on Day 4; therefore, the BAL fluid was collected on Day 4 in the subsequent experiment.

The mean fluorescence intensity (MIF) of CD206 in BAL macrophages in the LPS + MCTR1 group was enhanced compared with that in the LPS group (*P* < .05; Figure 2C). The expression of three M2 markers, Arg‐1, Ym‐1 and Relmα, in lung tissues was increased in the LPS + MCTR1 group compared with that of the LPS group (*P* < .05; Figure 2D‐G).

### 3.3 MCTR1 promoted M2 polarization of resident but not recruited macrophages

**FIGURE 3:**
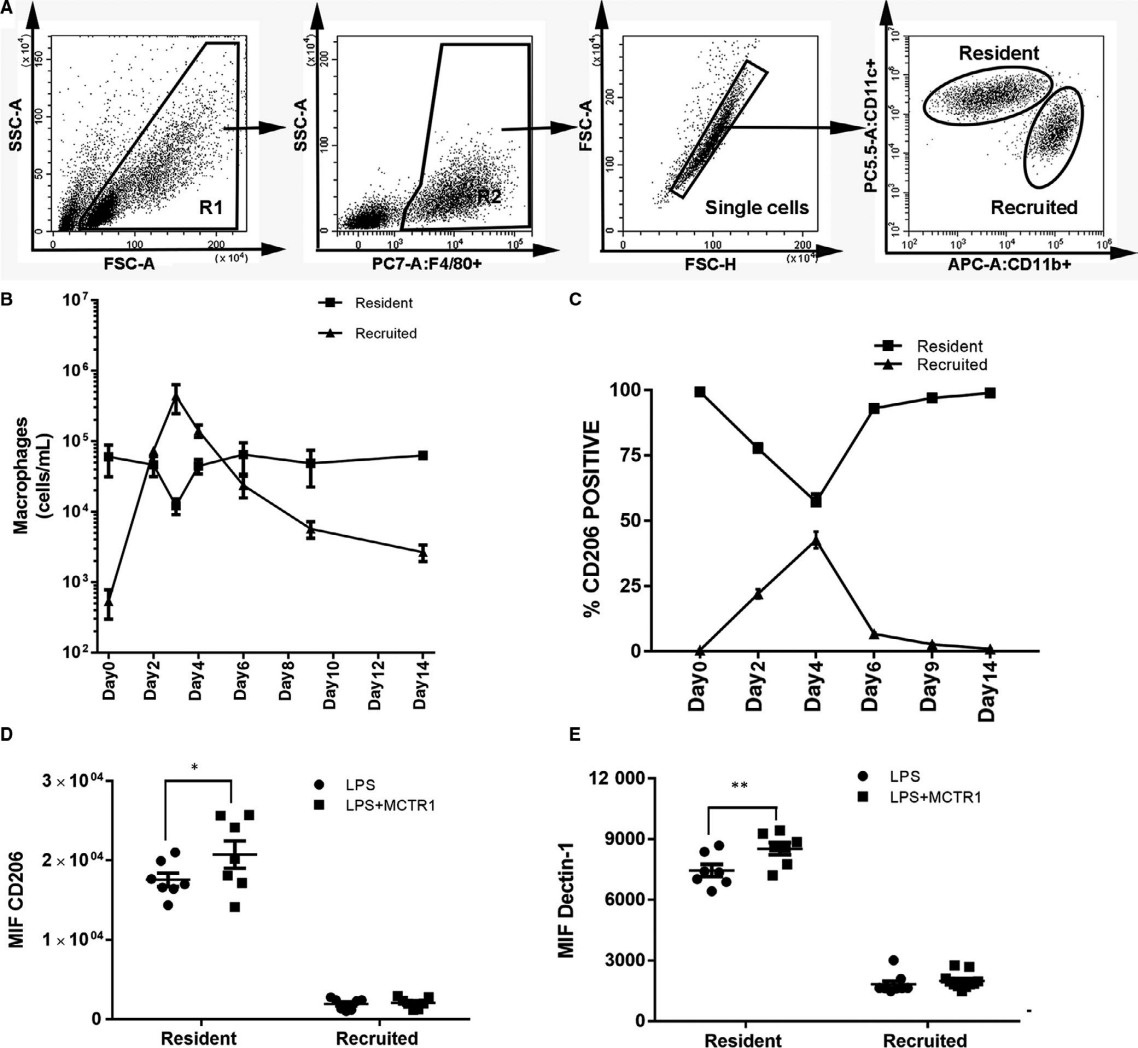
MCTR1 promoted M2 polarization of resident but not recruited macrophages. Macrophages in the BAL fluid were sorted by flow cytometry after 1 mg/kg LPS administration by inhalation (A). F4/80^+^CD11b^‐^CD11c^+^ resident macrophages and F4/80^+^CD11b^+^CD11c^−^ recruited macrophages in BAL fluid were counted by flow cytometry (B), and CD206‐positive cells (M2 macrophages) were also measured in these two macrophage populations on Day 0, Day 2, Day 4, Day 9 and Day 14 (C). Then, MCTR1 (0.1 µg/mouse) was administered 3 d after LPS (1 mg/kg) stimulation, and the MIF of CD206 and Detin‐1 (M2 marker) in the two populations of macrophages in the BAL fluid on Day 4 was measured (D, E). FSC‐A = forward scatter area, cell size. FSC‐H = forward scatter high, cell size. SSC‐A = side scatter area, cell shape. PC7‐A: F4/80 = F4/80‐PE‐Cyanine7 area. APC‐A:CD11b+=CD11b‐APC area. PC5.5‐A:CD11c = CD11c‐PerCP‐Cyanine5.5 area. The data are presented as the mean ± SEM. n = 6‐8. *P < .05, **P < .01

Resident and recruited macrophages were labelled with F4/80^+^CD11b^‐^CD11c^+^ and F4/80^+^CD11b^+^CD11c^‐^, respectively. We sorted resident and recruited macrophages from the BAL fluid of C57BL/6 mice (Figure 3A). The number of recruited macrophages increased after LPS stimulation, peaked on day 3 and then declined gradually to normal levels until Day 14 (Figure 3B). The resident macrophages remained largely constant during the course of inflammation (Figure 3B). CD206 was mainly expressed on resident macrophages, decreased during the first 2 days and then increased from Day 4 to Day 14 after LPS stimulation (Figure 3C). The percentage of CD206 cells among recruited macrophages increased after LPS stimulation and reached a maximum on Day 4 and then declined slowly to normal levels until Day 14 (Figure 3C).

In addition, compared with the LPS group, the MIFs of CD206 and Dectin‐1 on resident macrophages in the LPS + MCTR1 group were increased (*P* < .05), but there was no difference between the LPS and LPS + MCTR1 groups (*P* > .05; Figure 3D,E).

### 3.4 MCTR1 accelerated the resolution of inflammation via resident macrophages

**FIGURE 4:**
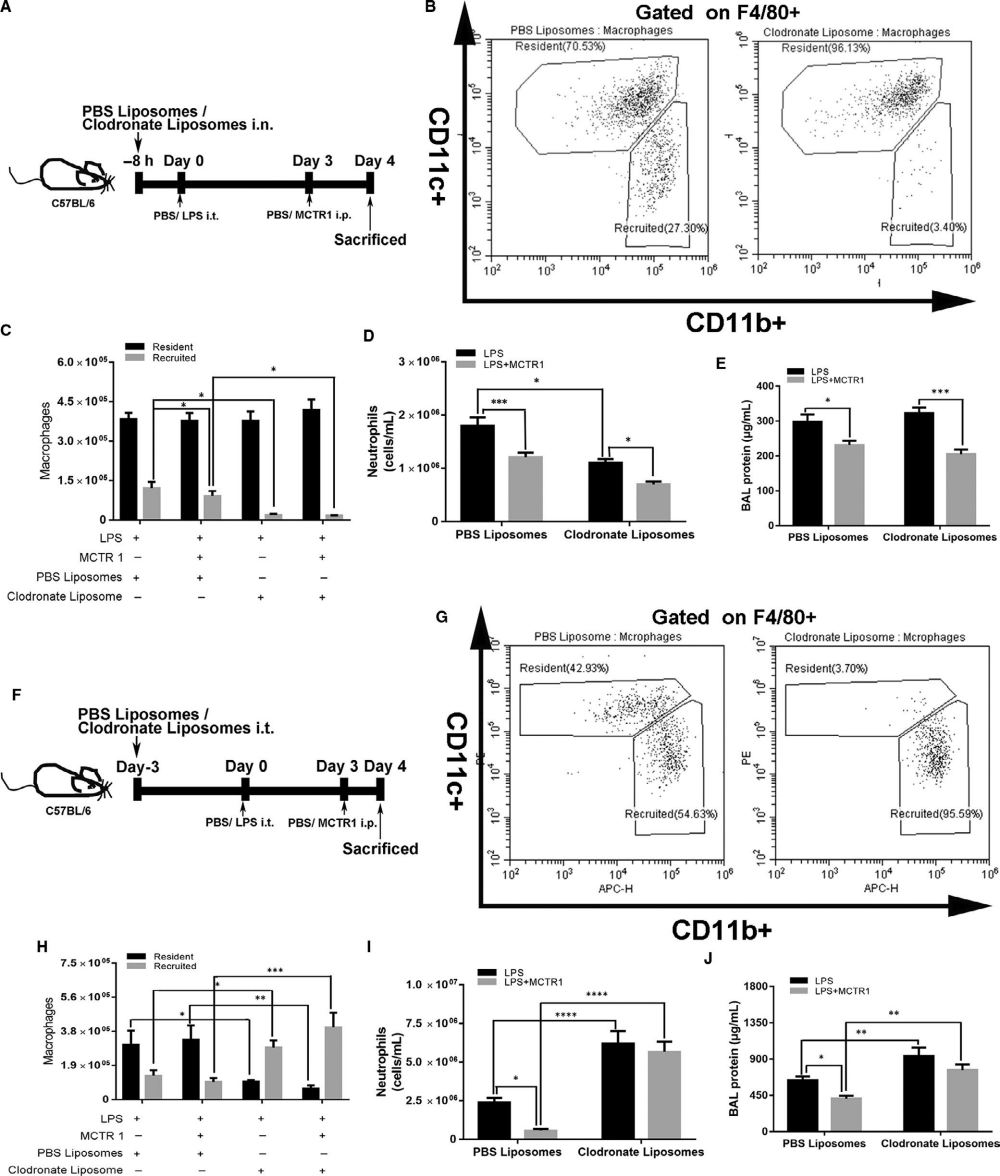
MCTR1 accelerated the resolution of inflammation by regulating resident macrophages. The different populations of macrophages were depleted by injecting clodronate liposomes, and then, MCTR1 (0.1 µg/mouse) was administered 3 d after LPS (1 mg/kg) stimulation. BAL fluid was collected on Day 4. The recruited macrophages were depleted by injecting 200 µL clodronate liposomes through the tail vein for 8 h (A, B), and then, macrophage (C), neutrophil (D) and protein levels (E) in the BAL fluid were detected. The resident macrophages were depleted by intratracheal administration of 50 µL clodronate liposomes for 3 d (E‐G), and then, the macrophage (H), neutrophil (I) and protein levels (J) in the BAL fluid were measured. The data are presented as the mean ± SEM. n = 6‐8. *P < .05, **P < .01, ***P < .001, ****P < .000

Recruited macrophages were depleted by clodronate liposome stimulation (Figure 4A,B). In the LPS + MCTR1+PBS liposome group, MCTR1 reduced the number of recruited macrophages compared with the LPS + PBS liposome group (*P* < .05; Figure 4C). The recruited macrophages were depleted by clodronate liposome intervention after LPS stimulation with or without MCTR1 (*P* < .05), indicating that our model was successfully established (Figure 4C). The neutrophil numbers and BAL protein reduced in the LPS + MCTR1 group compared with those of the LPS group after clodronate liposome administration to deplete recruited macrophages (*P* < .05; Figure 4D,E). MCTR1 also decreased BAL protein alone in the absence of clodronate liposomes, but MCTR1 was more effective in the presence of clodronate (Figure 4E).

In addition, after intratracheal clodronate liposome administration (Figure 4F,G), the resident macrophages were reduced compared with those of the PBS liposome group (*P* < .05; Figure 4H). The LPS + MCTR1+clodronate liposome group had fewer resident macrophages compared with those of the LPS + MCTR1+PBS liposome group (*P* < .05), suggesting that clodronate liposomes alone reduced the resident macrophages and that there was no additional effect of MCTR1 in this group (Figure 4H). Compared with the PBS liposome group, the neutrophil numbers and BAL protein were increased in the clodronate liposome group (*P* < .05; Figure 4I,J), but there were no significant differences between the LPS + MCTR1 and LPS groups after clodronate liposome stimulation (*P* > .05; Figure 4I,J).

### 3.5 Reinjected resident macrophages accelerated the resolution of inflammation

**FIGURE 5:**
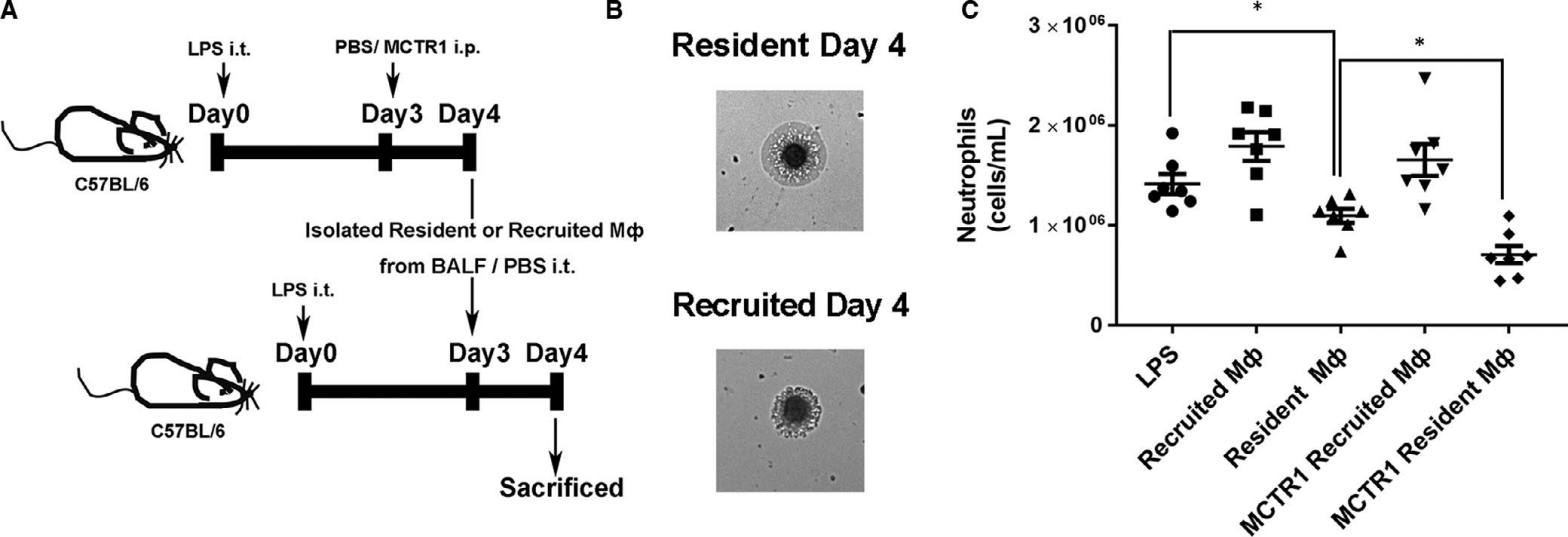
Reinjected resident macrophages accelerated the resolution of inflammation. Sorted resident or recruited macrophages on Day 4 were reinjected into the lung (A, B) to measure the neutrophil count by flow cytometry (C). The data are presented as the mean ± SEM. n = 6‐8. *P < .05, **P < .01, ***P < .001, ****P < .000

Furthermore, sorted resident or recruited macrophages were reinjected into the lung (Figure 5A). The resident macrophages exhibited a smooth nuclear shape and morphology similar to that of mature macrophages, and the recruited macrophages had irregular contours and morphology, resembling less mature macrophages (Figure 5B). Neutrophils were reduced in the resident Mø group compared with those in the LPS group (*P* < .05), and neutrophils were decreased in the MCTR1 resident Mø group compared with those in the resident Mø group (*P* < .05; Figure 5C). However, there were no significant changes in neutrophil numbers in the LPS, recruited Mø or MCTR1‐recruited Mø groups (*P* > .05; Figure 5C).

### 3.6 MCTR1 promoted M2 macrophage polarization via the STAT6 pathway, not IL‐4 and IL‐13

**FIGURE 6:**
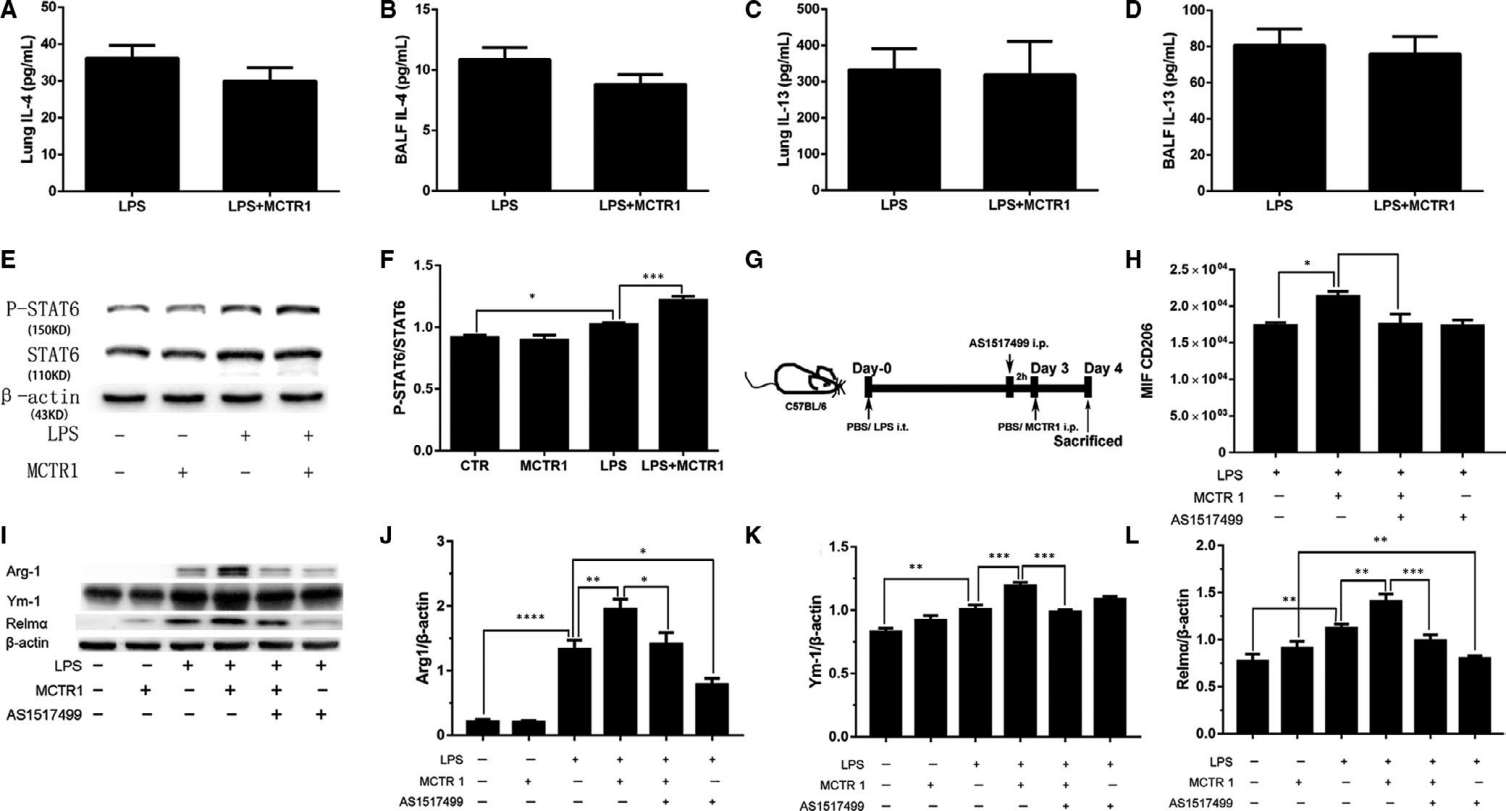
MCTR1 promoted M2 macrophage polarization via the STAT6 pathway, not IL‐4 and IL‐13. MCTR1 (0.1 µg/mouse) was administered 3 d after LPS (1 mg/kg) stimulation, and the lung tissues and BAL fluid were collected on Day 4. Then, IL‐4 (A, B) and IL‐13 (C, D) concentrations were detected by ELISA. Resident macrophages were sorted by flow cytometry. P‐STAT6 and STAT6 protein expression in resident macrophages was measured by Western blotting (E, F). Furthermore, AS1517499 (10 mg/kg), a STAT6 antagonist, was administered 2 h before MCTR1 (0.1 µg/mouse) treatment (G), and CD206 levels were measured by flow cytometry (H). Arg‐1, Ym‐1 and Relmα protein expression (M2 markers) was measured by Western blotting (I‐L). The data are presented as the mean ± SEM. n = 6‐8. *P < .05, **P < .01, ***P < .001, ****P < .000

It is well established that IL‐4 and IL‐13 play central roles in M2 macrophage polarization. However, in the present study, we found that there was no significant difference in IL‐4 and IL‐13 concentrations in lung tissues or BAL fluid between the LPS and LPS + MCTR1 groups (*P* > .05; Figure 6A‐D). However, we found that LPS administration increased p‐STAT6 protein levels in resident macrophages compared with those of the CTR group (*P* < .05; Figure 6E,F). Moreover, p‐STAT6 protein expression in resident macrophages was increased in the LPS + MCTR1 group compared with that of the LPS group (*P* < .05; Figure 6E,F). The increased protein level of CD206, Arg‐1, Ym‐1 and Relmα in resident macrophages in the BAL in the LPS + MCTR1 group was abrogated by AS1517499, a STAT6 inhibitor (*P* < .05; Figure 6G‐L).

### 3.7 The pro‐resolution effect of MCTR1 on lung injury inflammation was abolished by a STAT6 inhibitor

**FIGURE 7:**
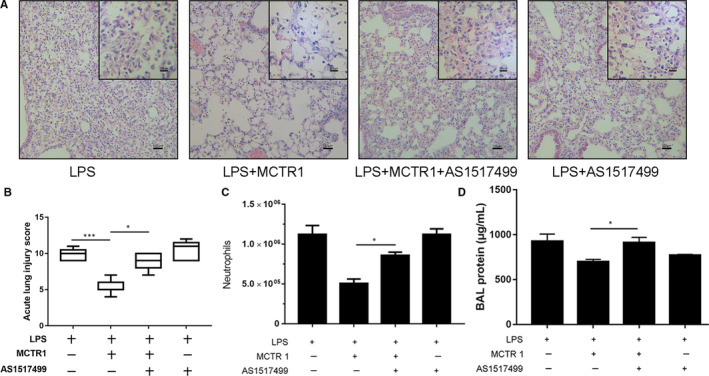
The pro‐resolution effect of MCTR1 on lung injury inflammation was abrogated by the STAT6 inhibitor. The mice received MCTR1 (0.1 µg/mouse) in the presence or absence of AS1517499 (10 mg/kg) via intraperitoneal injection 3 d after LPS administration. The effects of MCTR1 were assessed on Day 4 by (A) haematoxylin and eosin staining (original magnification: ×100, ×400), acute lung injury score (B), neutrophil numbers (C) and BAL protein (D) expression. The data are presented as the mean ± SEM. n = 6‐8. *P < .05, **P < .01, ***P < .001, ****P < .000

The benefit effect of MCTR1 on morphological changes was abolished by AS1517499 (Figure 7A). The acute lung injury score in the LPS + MCTR1+AS1517499 group was higher than that in the LPS + MCTR1 group (*P* < .05; Figure 7B). The neutrophil numbers were much higher in the LPS + MCTR1+AS1517499 group than in the LPS + MCTR1 group (*P* < .05; Figure 7C). The BFA protein concentration in the LPS + MCTR1+AS1517499 group was increased compared with the LPS + MCTR1 group (*P* < .05; Figure 7D).

## 4 DISCUSSION

In this study, we found that post‐treatment with MCTR1 on Day 3, at peak inflammation, decreased neutrophil numbers, BAL protein and albumin in mice 4 days after LPS‐induced lung injury. In addition, MCTR1 markedly promoted resident M2 macrophage polarization to accelerate the resolution of inflammation. In addition, MCTR1 increased STAT6 phosphorylation. AS1517499, a STAT6 inhibitor, abolished resident M2 macrophage polarization and the protective effects of MCTR1. These results indicate that MCTR1 promotes resident M2 macrophage polarization to accelerate the resolution of inflammation and was mainly mediated by the STAT6 pathway.

Resolution of inflammation is important for patient survival.^27^ Resolution of inflammation is characterized by the release of pro‐resolving mediators and the removal of infiltrated leucocytes.^28^, ^29^ In the present study, we introduced a set of resolution indices to define Ri as the time interval from the recorded maximum neutrophil infiltration point to the 50% reduction point.^30^, ^31^ We showed that Ri was 20 hours in an LPS‐induced lung injury model. After treatment with MCTR1, the Ri was 13 hours, suggesting that MCTR1 shortened the Ri. MCTR1 also reduced BAL protein and albumin, attenuated lung injury and enhanced mouse weight recovery 24 hours later. MCTR1 obviously promoted the resolution of LPS‐induced ARDS.

It is well known that macrophages act as critical effectors that induce inflammation. In ARDS, macrophages, including M1 (pro‐inflammatory) and M2 (anti‐inflammatory) macrophages, are present in the BAL fluid. M2 macrophage polarization is a key step in the resolution of ARDS inflammation.^32^, ^33^ Here, we demonstrated that the total number of macrophages peaked on Day 3 and then gradually declined, but the percentage of M2 macrophages decreased during the first 2 days and then gradually increased until Day 14. Treatment with MCTR1 at the peak of inflammation up‐regulated CD206, Arg‐1, Ym‐1 and Relmα protein expression in LPS‐induced lung injury, indicating that MCTR1 promoted M2 polarization of macrophages.

Two populations of macrophages, resident alveolar macrophages and recruited macrophages from circulating monocytes, participate in the pathogenesis of ARDS. Next, we investigated the effects of MCTR1 on M2 polarization of the two types of macrophages. We found that recruited macrophages^26^ were increased and peaked on Day 3 and then recovered to the steady state on Day 14 after LPS exposure, but resident alveolar macrophages^26^ were stabilized during these days, suggesting that after LPS stimulation, a large number of recruited macrophages infiltrate the lung to participate in initiating inflammation and then are reduced in the resolution phase, but the number of resident alveolar macrophages changed very little, which was consistent with a previous study.^34^ The percentage of CD206 cells decreased among resident macrophages until Day 4 and then rose back to a level similar to that of Day 0 from days 6‐14 after LPS stimulation, suggesting that M2 macrophages were mainly derived from resident macrophages. In addition, we also found that treatment with MCTR1 up‐regulated the MIF of CD206 and Dectin‐1 expression on resident macrophages but not recruited macrophages, suggesting that M2 macrophages mainly originated from resident macrophages during the resolution phase and that MCTR1 promoted resident M2 polarization.

We further investigated the effects of MCTR1 on the two populations of macrophages. Clodronate liposomes were used to deplete the different populations of macrophages. MCTR1 also decreased neutrophil numbers and BAL protein expression when recruited macrophages were depleted. However, MCTR1 had no effect on neutrophil numbers or BAL protein expression when resident macrophages were depleted. These results indicated that MCTR1 accelerated the resolution of inflammation through resident macrophages.

To further clarify the importance of resident macrophages in the resolution phase, sorted resident and recruited macrophages were reinjected into the lung. We found that neutrophil numbers were decreased after resident but not recruited macrophages were reinjected into the lung. In addition, the neutrophils decreased much more in the MCTR1 treatment with resident macrophage group. These results suggest that MCTR1 promotes resident M2 macrophage polarization to accelerate the resolution of inflammation.

The mechanisms by which MCTR1 promote resident M2 macrophage polarization to accelerate inflammation resolution in mice remain to be clarified. A previous study showed that IL‐4 and IL‐13 are involved in macrophage polarization,^3^, ^35^ but we found that the IL‐4 and IL‐13 levels were unchanged after treatment with MCTR1 in LPS‐induced mice, indicating that MCTR1 regulates macrophage polarization to promote the resolution of inflammation independent of IL‐4 and IL‐13.

Activation of STAT6 is a key signalling pathway in macrophage function. Recent work has demonstrated that STAT6‐dependent M2 protein upregulation is important in accelerating resolution in LPS‐induced lung injury.^3^ STAT6 activation can improve the FIZZ1, Ym‐1 and arginase level, thus enhancing the differentiation of M2.^36^, ^37^ Consistently, we found that treatment with MCTR1 increased STAT6 phosphorylation without affecting total STAT6 expression in resident macrophages. The MCTR1‐mediated increase in CD206, Arg‐1, Ym‐1 and Relmα protein expression in resident macrophages and protection against lung injury were abrogated by the STAT6 inhibitor AS1517499, suggesting that MCTR1‐promoted resident M2 macrophage polarization was dependent on the STAT6 pathway.

In summary, MCTR1 decreased neutrophil numbers and BAL protein and albumin concentrations, enhanced inflammation resolution and attenuated lung injury by promoting resident M2 macrophage polarization, which was mediated by the STAT6 pathway. Our findings reveal that MCTR1 may be a new treatment strategy for ARDS.

## CONFLICT OF INTEREST

The other authors declared no conflict of interest.

## **AUTHOR** **CONTRIBUTIONS**

Qian Wang and Hua‐Wei Zhang designed the research study and performed the research. Hong‐Xia Mei, Yang Ye, Hao‐Ran Xu and Shu‐Yang Xiang collated the data and carried out data analyses. Qian Yang, Sheng‐Xing Zheng and Fang Gao Smith contributed to drafting the manuscript. Sheng‐Wei Jin revised the paper. All authors have read and approved the final submitted manuscript.

## Data Availability

I confirm that the article contains a Data Availability Statement even if no data are available. I confirm that I have included a citation for available data in my references section.

## References

[1] Ware LB , Matthay MA . The acute respiratory distress syndrome. N Engl J Med. 2000;342:1334‐1349. 10793167 10.1056/NEJM200005043421806

[2] Hamid U , Krasnodembskaya A , Fitzgerald M , et al. Aspirin reduces lipopolysaccharide‐induced pulmonary inflammation in human models of ARDS. Thorax. 2017;72:971‐980. 28082531 10.1136/thoraxjnl-2016-208571 PMC5858553

[3] D'Alessio FR , Craig JM , Singer BD , et al. Enhanced resolution of experimental ARDS through IL‐4‐mediated lung macrophase reprogramming. Am J Physiol Lung Cell Mol Physiol. 2016;310:733‐746. 10.1152/ajplung.00419.2015 PMC4836113 26895644

[4] Aggarwal NR , Tsushima K , Eto Y , et al. Immunological priming requires regulatory T cells and IL‐10‐producing macrophages to accelerate resolution from severe lung inflammation. J Immunol. 2014;192:4453‐4464. 24688024 10.4049/jimmunol.1400146 PMC4001810

[5] Gordon S , Taylor PR . Monocyte and macrophage heterogeneity. Nat Rev Immunol. 2005;5:953‐964. 16322748 10.1038/nri1733

[6] Mantovani A , Biswas SK , Galdiero MR , et al. Macrophage plasticity and polarization in tissue repair and remodelling. J Pathol. 2013;229:176‐185. 23096265 10.1002/path.4133

[7] Sica A , Mantovani A . Macrophage plasticity and polarization: in vivo veritas. J Clin Invest. 2012;122:787‐795. 22378047 10.1172/JCI59643 PMC3287223

[8] Hashimoto D , Chow A , Noizat C , et al. Tissue‐resident macrophages self‐maintain locally throughout adult life with minimal contribution from circulating monocytes. Immunity. 2013;38:792‐804. 23601688 10.1016/j.immuni.2013.04.004 PMC3853406

[9] Yona S , Kim K‐W , Wolf Y , et al. Fate mapping reveals origins and dynamics of monocytes and tissue macrophages under homeostasis. Immunity. 2013;38:79‐91. 23273845 10.1016/j.immuni.2012.12.001 PMC3908543

[10] Zasłona Z , Przybranowski S , Wilke C , et al. Resident alveolar macrophages suppress, whereas recruited monocytes promote, allergic lung inflammation in murine models of asthma. J Immunol. 2014;193:4245‐4253. 25225663 10.4049/jimmunol.1400580 PMC4185233

[11] Mould KJ , Barthel L , Mohning MP , et al. Cell origin dictates programming of resident versus recruited macrophages during acute lung injury. Am J Respir Cell Mol Biol. 2017;57:294‐306. 28421818 10.1165/rcmb.2017-0061OC PMC5625228

[12] Hussell T , Bell TJ . Alveolar macrophages: plasticity in a tissue‐specific context. Nat Rev Immunol. 2014;14:81‐93. 24445666 10.1038/nri3600

[13] Sindrilaru A , Peters T , Wieschalka S , et al. An unrestrained proinflammatory M1 macrophage population induced by iron impairs wound healing in humans and mice. J Clin Invest. 2011;121:985‐997. 21317534 10.1172/JCI44490 PMC3049372

[14] Gordon S , Martinez FO . Alternative activation of macrophages: mechanism and functions. Immunity. 2010;32:593‐604. 20510870 10.1016/j.immuni.2010.05.007

[15] Arnold L , Henry A , Poron Françoise , et al. Inflammatory monocytes recruited after skeletal muscle injury switch into antiinflammatory macrophages to support myogenesis. J Exp Med. 2007;204:1057‐1069. 17485518 10.1084/jem.20070075 PMC2118577

[16] Nahrendorf M , Swirski FK , Aikawa E , et al. The healing myocardium sequentially mobilizes two monocyte subsets with divergent and complementary functions. J Exp Med. 2007;204:3037‐3047. 18025128 10.1084/jem.20070885 PMC2118517

[17] Aggarwal NR , King LS , D’Alessio FR . Diverse macrophage populations mediate acute lung inflammation and resolution. Am J Physiol Lung Cell Mol Physiol. 2014;306:709‐725. 10.1152/ajplung.00341.2013 PMC3989724 24508730

[18] Herold S , Mayer K , Lohmeyer J . Acute lung injury: how macrophages orchestrate resolution of inflammation and tissue repair. Front Immunol. 2011;2:65. 22566854 10.3389/fimmu.2011.00065 PMC3342347

[19] Dalli J , Sanger JM , Rodriguez AR , et al. Identification and actions of a novel third maresin conjugate in tissue regeneration: MCTR3. PLoS One. 2016;11:e0149319. 26881986 10.1371/journal.pone.0149319 PMC4755597

[20] Dalli J , Chiang N , Serhan CN . Identification of sulfido‐conjugated mediators that promote resolution of infection and organ protection. Proc Natl Acad Sci USA. 2014;111:4753‐4761. 10.1073/pnas.1415006111 PMC4226123 25324525

[21] Dalli J , Ramon S , Norris PC , et al. Novel proresolving and tissue‐regenerative resolvin and protectin sulfido‐conjugated pathways. FASEB J. 2015;29:2120‐2136. 25713027 10.1096/fj.14-268441 PMC4415017

[22] Serhan CN . Pro‐resolving lipid mediators are leads for resolution physiology. Nature. 2014;510:92‐101. 24899309 10.1038/nature13479 PMC4263681

[23] Dalli J , Vlasakov I , Riley IR , et al. Maresin conjugates in tissue regeneration biosynthesis enzymes in human macrophages. Proc Natl Acad Sci USA. 2016;113:12232‐12237. 27791009 10.1073/pnas.1607003113 PMC5087052

[24] Dalli J , Serhan CN . Pro‐resolving mediators in regulating and conferring macrophage function. Front Immunol. 2017;8:1400. 29163481 10.3389/fimmu.2017.01400 PMC5671941

[25] Chiang N , Riley IR , Dalli J , et al. New maresin conjugates in tissue regeneration pathway counters leukotriene D‐stimulated vascular responses. FASEB J. 2018;32:4043‐4052. 29490167 10.1096/fj.201701493R PMC5998978

[26] Ueno M , Maeno T , Nishimura S , et al. Alendronate inhalation ameliorates elastase‐induced pulmonary emphysema in mice by induction of apoptosis of alveolar macrophage. Nat Commun. 2015;6:6332. 25757189 10.1038/ncomms7332

[27] Sznajder JI . Alveolar edema must be cleared for the acute respiratory distress syndrome patient to survive. Am J Respir Crit Care Med. 2001;163:1293‐1294. 11371384 10.1164/ajrccm.163.6.ed1801d

[28] Sugimoto MA , Ribeiro ALC , Costa BRC , et al. Plasmin and plasminogen induce macrophage reprogramming and regulate keysteps of inflammation resolution via annexin A1. Blood. 2017;129:2896‐2907. 28320709 10.1182/blood-2016-09-742825 PMC5445571

[29] Sugimoto MA , Sousa LP , Pinho V , et al. Resolution of Inflammation. Front Immunol. 2016;7:160. 27199985 10.3389/fimmu.2016.00160 PMC4845539

[30] Bannenberg GL , Chiang N , Ariel A , et al. Molecular circuits of resolution: formation and actions of resolvin and protectins. J Immunol. 2005;174:4345‐4355. 15778399 10.4049/jimmunol.174.7.4345

[31] Navarro‐Xavier RA , Newson J , Silveira VL , et al. A new strategy for the identification of novel molecules with targeted proresolution of inflammation properties. J Immunol. 2010;184:1516‐1525. 20032295 10.4049/jimmunol.0902866

[32] Misharin AV , Scott Budinger GR , Perlman H . The lung macrophage: a Jack of all trades. Am J Respir Crit Care Med. 2011;184:497‐498. 21885631 10.1164/rccm.201107-1343ED PMC3175549

[33] Ji WJ , Ma YQ , Zhang X , et al. Inflammatory monocyte/macrophage modulation by liposome‐entrapped spironolactone ameliorates acute lung injury in mice. Nanomedicine (Lond). 2016;11:1393‐1406. 27221077 10.2217/nnm-2016-0006 PMC5561989

[34] Schiwon M , Weisheit C , Franken L , et al. Crosstalk between sentinel and helper macrophages permits neutrophil migration into infected uroepithelium. Cell. 2014;156:456–468. 24485454 10.1016/j.cell.2014.01.006 PMC4258064

[35] Schmieder A , Michel J , Schönhaar K , et al. Differentiation and gene expression profile of tumor‐associated macrophages. Semin Cancer Biol. 2012;22:289‐297. 22349514 10.1016/j.semcancer.2012.02.002

[36] Mikita T , Campbell D , Wu P , et al. Requirements for interleukin‐4‐induced gene expression and functional characterization of Stat6. Mol Cell Biol. 1996;16:5811‐5820. 8816495 10.1128/mcb.16.10.5811 PMC231582

[37] Moreira AP , Cavassani KA , Hullinger R , et al. Serum amyloid P attenuates M2 macrophage activation and protects against fungal spore‐induced allergic airway disease. J Allergy Clin Immunol. 2010;126:712‐721. 20673988 10.1016/j.jaci.2010.06.010

